# Outbursts from an ice-marginal lake in Antarctica in 1969–1971 and 2017, revealed by aerial photographs and satellite data

**DOI:** 10.1038/s41598-023-47522-w

**Published:** 2023-11-27

**Authors:** Shuntaro Hata, Moto Kawamata, Koichiro Doi

**Affiliations:** 1 Institute of Low Temperature Science, Hokkaido University, Sapporo, Japan; 2 Creative Research Institution, Hokkaido University, Sapporo, Japan; 3 Civil Engineering Research Institute for Cold Region, Public Works Research Institute, Sapporo, Japan; 4 National Institute of Polar Research, Tokyo, Japan; 5 The Graduate University for Advanced Studies (SOKENDAI), Hayama, Japan

**Keywords:** Cryospheric science, Hydrology, Limnology

## Abstract

The liquid water around the Antarctic Ice Sheet plays a key role in modulating both the vulnerability of ice shelves to hydrofracturing and ice discharge from outlet glaciers. Therefore, it needs to be adequately constrained for precise future projections of ice-mass loss and global sea-level rise. Although glacial lake outburst floods (GLOFs) pose one of the greatest risks in glacierized mountainous regions, any long-term monitoring of Antarctic ice-marginal lakes and their associated potential for GLOFs has been neglected until recently owing to the limited number of such events reported in Antarctica. Here we present direct evidence of repeated GLOFs from Lake Kaminotani-Ike, an ice-sheet-dammed lake in East Antarctica, via an analysis of historical aerial photographs and recent satellite data. Two GLOFs occurred in 1969–1971 and 2017, with discharge volumes of (8.6 ± 1.5) × 10^7^ and (7.1 ± 0.4) × 10^7^ m^3^, respectively, making them two of the largest GLOFs in Antarctica. A southerly oceanward pathway beneath the ice sheet is the most likely drainage route of these GLOF events based on the available surface- and bed-elevation datasets. Furthermore, the 2017 event occurred during the austral winter, thereby implying the possibility of year-round active subglacial networks in Antarctica. Our results highlight that studies on Antarctic ice-marginal lakes provide an opportunity to better understand Antarctic hydrological processes and emphasize the need for both detailed monitoring of ice-marginal lakes and detailed surveying of the subglacial environments of the Antarctic Ice Sheet.

## Introduction

The Antarctic Ice Sheet (AIS) is the largest potential contributor to sea-level rise^1, 2^. Recent studies have revealed that the hydrology of Antarctica plays a key role in sea-level rise by modulating the ice dynamics of AIS outlet glaciers and ice shelves^3–7^. The ongoing increase in supraglacial lakes has the potential to destabilize ice shelves via hydrofracturing^3, 7, 8^, although supraglacial streams can weaken this process by reducing lake storage via water transport^9^. Subsequent acceleration of the inland ice may occur if the ice shelf collapses^10–12^ and/or meltwater drainage reaches the subglacial environment^3–5^. Outburst floods from subglacial lakes have accelerated the downglacier flow of outlet glaciers by altering the subglacial hydrological environment^5, 13^. However, the hydrological processes behind such events are not well constrained because of the remoteness of the subglacial environment. Despite the importance of cryo-hydrology in the evolution of AIS, studies of ice-marginal and/or ice-dammed lakes are extremely limited, thereby limiting our understanding of AIS hydrology.

Glacial lake outburst floods (GLOFs), which abruptly discharge their stored water and lake materials downstream, are a common phenomenon that has been observed at ice-marginal lakes in glacierized regions worldwide, with the exception of Antarctica, and are recognized as one of the greatest risks in mountain societies^14–17^. Furthermore, these freshwater pulses can impact ocean circulation, biochemical circulation, and marine ecosystems^18–20^. For example, GLOFs from the largest-scale glacial lakes that were located along the periphery of past ice sheets weakened ocean circulation, thereby modulating climate^21, 22^. However, GLOFs from ice-marginal and ice-dammed lakes in Antarctica have largely been neglected due to extremely limited reports of GLOFs until recently^23–26^. Although outbursts from subglacial, supraglacial, and sub-surface lakes are generally associated with either a collapse of the surrounding ice or ice doline formation^27–29^, the subglacial and englacial hydrological processes behind such events are not well constrained due to the rarity of these events and the inherent difficulty in obtaining direct observations. The lack of ice-marginal lake monitoring in Antarctica limits our understanding of the impacts that GLOFs have on ice-sheet/glacier dynamics and the surrounding environment. Furthermore, constraints on GLOFs in Antarctica may advance our understanding of lake–ice interactions in harsh environments, even though they pose a lower risk compared to other regions.

**Figure 1:**
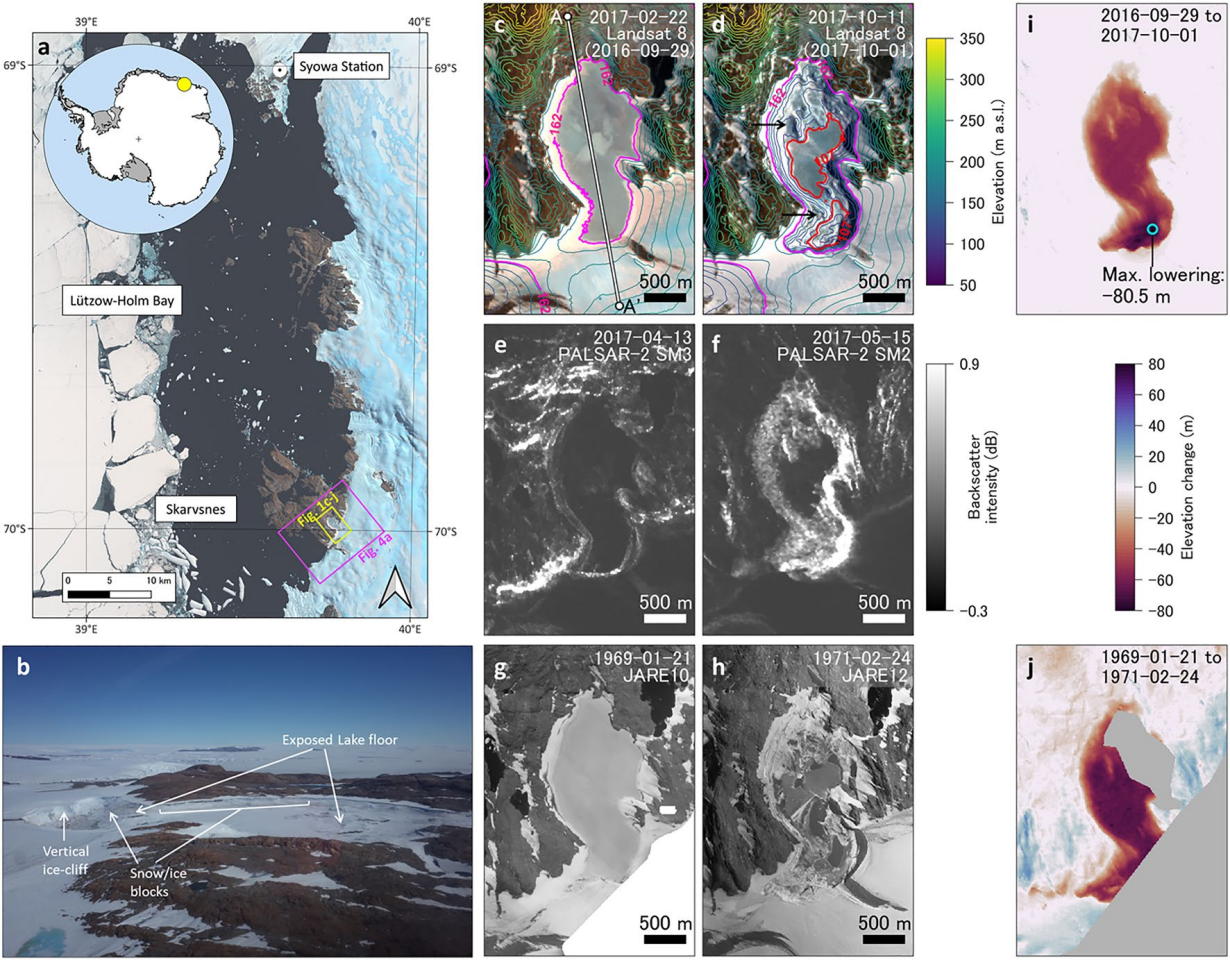
Overview of the LKI GLOFs. (**a**) Overview of the study region. The background image is a Landsat 8 satellite image that was taken on 22 February 2017. The inset map shows the location of the study region relative to Antarctica. (**b**) Field photographs taken on 10 January 2018, after the 2017 LKI GLOF. (**c**, **d**) Landsat 8 satellite images taken on (**c**) 22 February and (**d**) 11 October 2017. The contours were taken from the REMA-DEMs on (**c**) 29 September 2016 and (**d**) 1 October 2017. Black arrows highlight the location of the exposed lake floor shown in (**b**). (**e**, **f**) PALSAR-2 satellite backscatter intensity images taken on (**e**) 13 April and (f) 15 May 2017. (**g**, **h**) Orthoimages generated from aerial photographs taken on (**g**) 21 January 1969 and (**h**) 24 February 1971. Surface-elevation changes during the (**i**) 29 September 2016 to 1 October 2017 and (**j**) 21 January 1969 to 24 February 1971 periods.

Lake Kaminotani-Ike (LKI) is an ice-dammed (and perennially ice-covered ice-marginal) lake in East Antarctica. LKI is situated ~ 60 km south of Syowa Station and is located between the Skarvsnes outcrop and the East AIS (EAIS; Fig. 1a). Liquid precipitation rarely occurs across the Lützow-Holm Bay region. For example, a rain event on 24 December 2017 at Syowa Station marked the first recorded since 2012^30^. Therefore, the lake water likely originates from subglacial discharge that is directly fed by EAIS and meltwater of solid precipitation over the catchment. Members of the 59th Japanese Antarctic Research Expedition (JARE59) observed several unusual features at LKI on 22 November 2017, including the exposed lake floor, a vertical ice cliff at the boundary between the lake and EAIS, and snow and ice blocks scattered along the lake shore^31^ (Fig. 1b). All of these features implied rapid surface lowering. Although information about LKI is limited, previous studies have suggested the possibility of dynamic changes at LKI^32, 33^ and captured details on the drainage and periodicity of a nearby ice/snow-dammed lake^23, 24^.

Aerial photographs had been acquired across Lützow-Holm Bay, East Antarctica, by JARE since 1957 (Supplementary Fig. 1). The photographs had been taken across the Skarvsnes region during the austral summer over the 1957–2000 period. Development of SfM techniques enable us to trace surface elevation by constructing digital elevation models (DEMs) from historical aerial photographs. This aerial photograph collection, coupled with recent satellite data (imagery and DEMs), provides a ~ 60-year time series of the surface state and surface elevation of LKI. In this paper, we present direct evidence of repeated GLOFs from an Antarctic ice-dammed lake and infer their potential periodicity via an analysis of long-term historical aerial photographs and recent satellite data.

## Results

### Discovery of outburst events

We found two GLOF events in 1969–1971 and 2017 from satellite imagery and aerial photographs (we hereafter refer to the GLOF periods as P1 and P2, respectively). A comparison of Landsat 8 satellite images acquired on 22 February and 11 October 2017 clearly highlighted changes in the surface features of LKI (Fig. 1c and d). The lake surface was flat due to frozen lake ice on 22 February (Fig. 1c), whereas on 11 October the surface was rugged and the lake-floor terrain was exposed (Fig. 1d), with the latter remote-sensing-based features supported by field observations (Fig. 1b). Furthermore, PALSAR-2 synthetic aperture radar (SAR) images acquired on 13 April and 15 May 2017 highlighted clear changes in both the lake-surface features and backscatter intensities between the two images (Fig. 1e and f). Although the lake surface was flat and possessed a uniform spatial distribution of backscatter intensities on 13 April 2017 (Fig. 1e), rugged features and an increase in backscatter intensity were observed at the periphery of the lake on 15 May 2017 (Fig. 1f). The 15 May PALSAR-2 image detected a lake shoreline with a similar shape to that in the 11 October 2017 optical image (Fig. 1d), which indicates that the drainage of P2 occurred sometime during the April–May 2017 period and was at most a one-month-long drainage event. We detected another LKI drainage event during the 1969–1971 period that exhibited similar surface feature changes to those associated with the 2017 GLOF event, whereby a flat surface was observed until 1969 and then became rugged, with the lake-floor terrain being exposed in 1971 (Fig. 1g and h). No other abrupt changes in the surface features of LKI have been observed in the aerial photographs and satellite images (Supplementary Fig. 2).

### Surface elevation change of LKI

**Figure 2:**
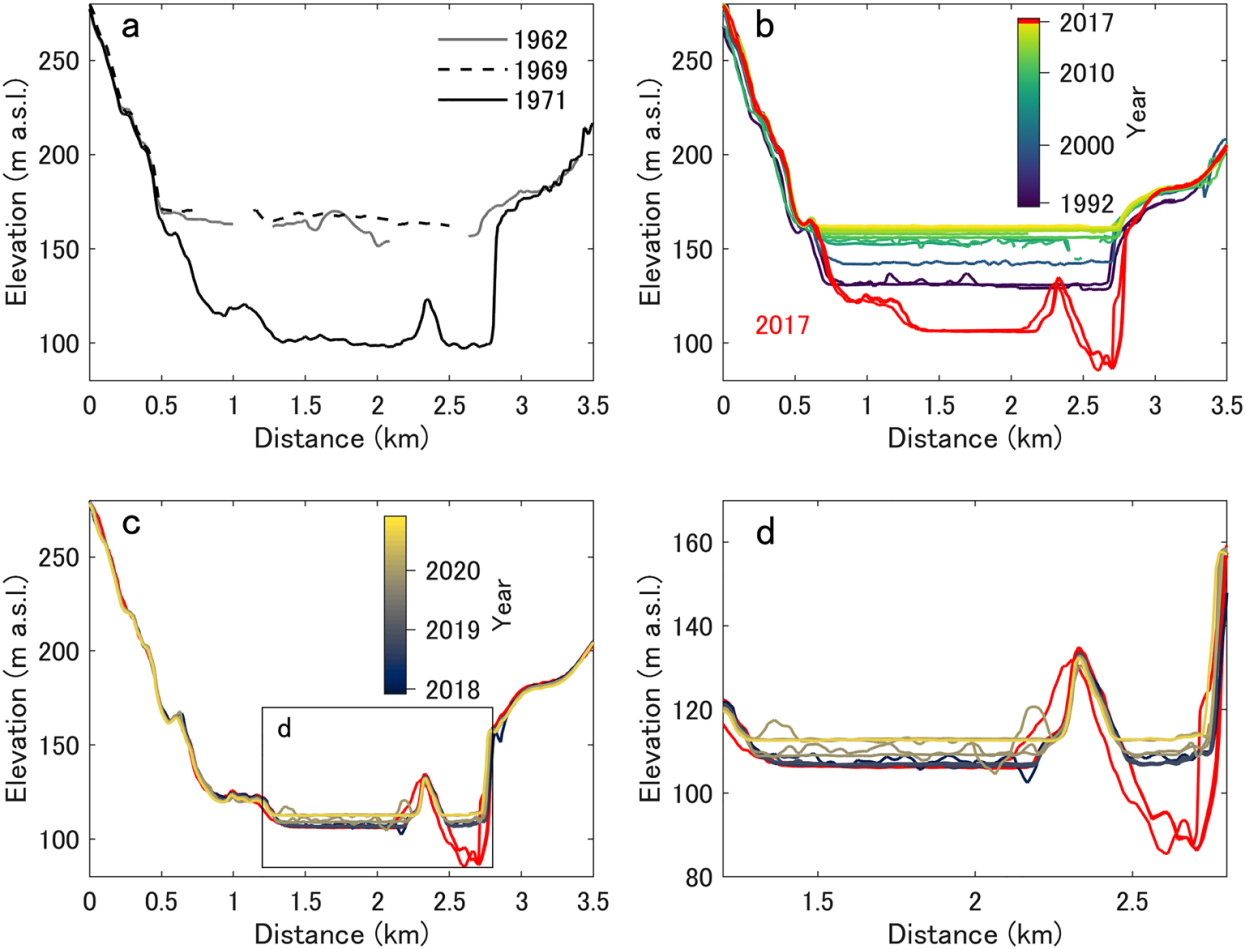
LKI elevation profiles. Surface elevation along profile AA′ (Fig. 1c), which crosses LKI, during the (**a**) 1962–1971, (**b**) 1992–2017, and (**c**) 2018–2020 periods. (**d**) Close-up view of (**c**) to concentrate on the lake-surface uplift after the 2017 GLOF. Note that the color schemes in (**b**) and (**c**) are different.

**Figure 3:**
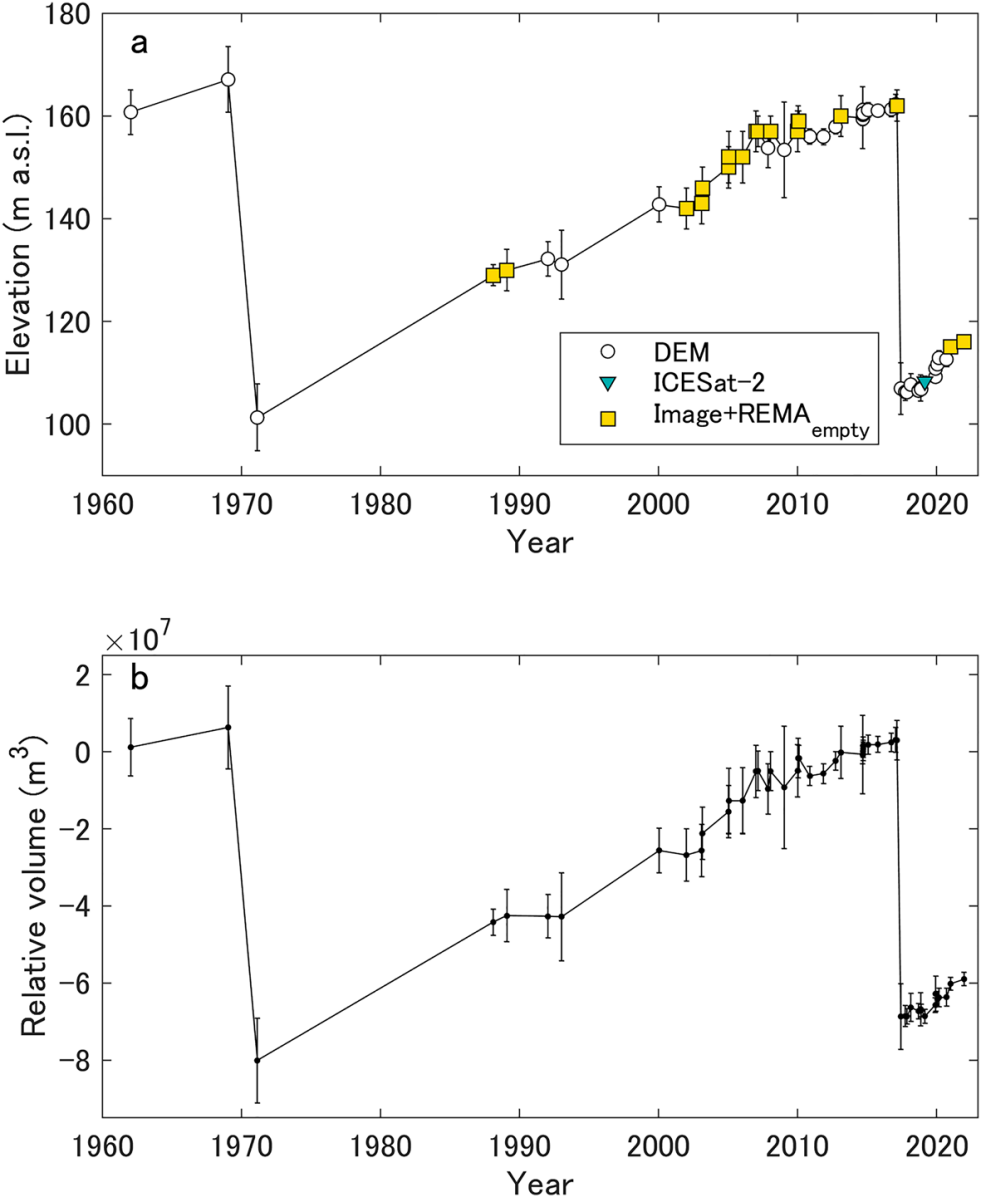
Lake-surface elevation and volume time series. Time series of (**a**) lake-surface elevation and (**b**) relative lake volume, which is referenced to the mean of the REMA period. The “DEM” data points are derived from the DEMs (SfM-, REMA-, GSI-, TDX-, and ALOS-DEMs). The “REMA_empty_ + Image” data points are derived from the lake shoreline method, which employed REMA_empty_ in (**a**). The “ICESat-2” data points are from the ICESat-2 ATL06 product (Supplementary Fig. 7).

We traced the LKI surface-elevation changes using satellite-based DEMs (REMA^34, 35^-, ALOS-, GSI-, and TDX-DEMs; see the Methods section) and DEMs that were constructed by applying the structure-from-motion (SfM) technique to the JARE aerial photographs (SfM-DEMs). DEMs clearly capture a drop in surface elevation across LKI in 1969–1971 and 2017 (Fig. 1c,d, and 2c and Supplementary Fig. 2). The surface elevation dropped by 65.8 ± 9.1 and 55.0 ± 2.1 m, which indicates discharge of (8.6 ± 1.5) × 10^7^ and (7.1 ± 0.4) × 10^7^ m^3^ from LKI during P1 and P2, respectively (Figs. 1i, j, 2, and 3). A maximum surface lowering of 80.5 ± 2.1 m was observed near EAIS front during P2 (Fig. 1i). This deepest part was not captured in the 1971 DEM, which suggests that either the exact drainage of P1 occurred long enough before the acquisition date (24 February 1971) for refilling to have already begun, or LKI may not have fully drained during P1 (Fig. 2). The total discharge volumes from LKI during P1 and P2 are four times larger than the 2019 GLOF event at Lake Untersee, a glacier-dammed lake in East Antarctica^25^, thereby making these LKI events two of the largest GLOFs reported at an ice-marginal lake in Antarctica to date.

We analyzed all of the available elevation datasets (aerial photography, satellite laser altimetry, and a combination of optical satellite imagery and a post-GLOF DEM) to construct a dense time series of the lake-surface elevation and relative lake volume (Fig. 3). The lake volume increased by 1.7 × 10^6^ m^3^ a^−1^ between P1 and P2 (the 1971–2017 period) and 2.5 × 10^6^ m^3^ a^−1^ after P2 (the 2017–2021 period). The time series clearly highlights two GLOFs that are followed by periods of gradual lake filling (Fig. 3a). The changes in lake-surface elevation and lake volume exhibit a typical saw-tooth pattern (Fig. 3) that is similar to the cyclic patterns of rapid drainage and slow filling that have been observed at ice-dammed lakes in other regions^36–38^.

## Discussion

### Outburst mechanism

Outbursts from ice-dammed lakes are generally caused by either a break in the seal (between the glacier bottom and bedrock) or overflow of the ice dam/lake margin. Seal-break mechanisms are classified as (1) flotation^36, 39^, (2) conduit formation beneath/within the ice dam^40^, and (3) mechanical collapse^41^. Evidence of these different outburst mechanisms have been observed in the field at Antarctic glacial lakes, even though Antarctic GLOF studies are limited. For example, the 2019 Untersee GLOF was initiated by water outflow at the lake margin, which was confirmed by field inspections^25^. The Yatsude Valley GLOF was probably caused via the subglacial hydrological system, with the formation of a near-circular hole at/near the base of the ice dam inferred^23^. There were no observations of a discernible hole, trace of failure in the ice dam, or surface-water overflow during the 2017 field inspection and in the images that were acquired after the 2017 LKI GLOF event. However, the lake-surface elevations immediately prior to the 1969–1971 and 2017 LKI GLOFs were quite similar. Therefore, we assumed that the triggering mechanism of the LKI GLOFs was related to the flotation criterion of the ice dam, as observed for GLOFs from other ice-dammed lakes^36, 42^. Even if the ice dam achieved flotation, the glacier uplift may not be detectable in the satellite-based DEMs due to the expected small-scale vertical motion of the ice dam (several centimeters)^39^.

**Figure 4:**
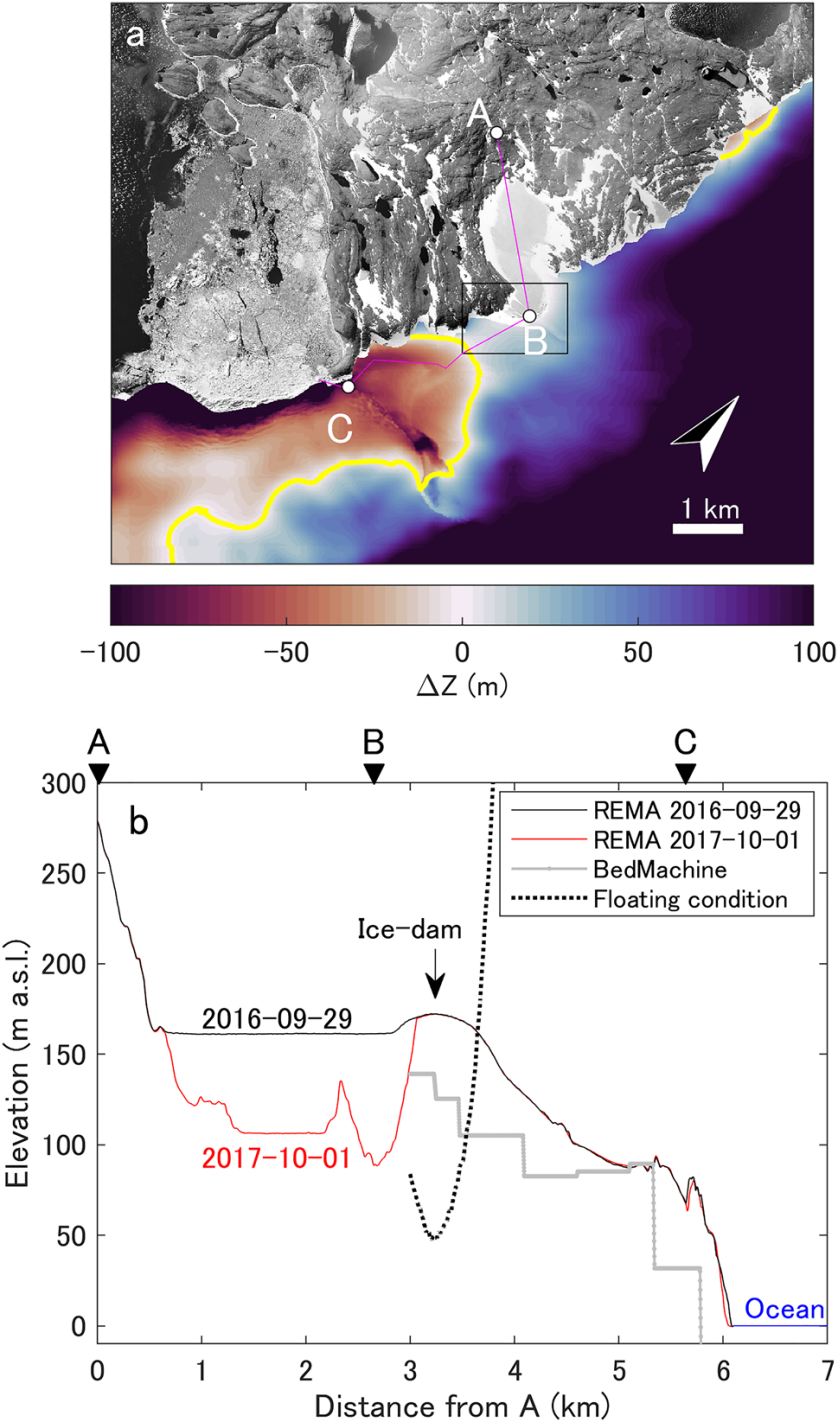
Estimation of discharge routing. (**a**) Spatial $\Delta z$ distribution, generated using the REMA-DEM on 29 September 2016 and the BedMachine version 3 dataset^43^. Yellow lines indicate where $\Delta z$ = 0. The background image is an orthoimage generated from aerial photographs taken on 19 January 2000. The box indicates the extent of Fig. 5. (**b**) Cross section of the surface and bed elevations along profile ABC, with the position of the profile shown in (**a**). Black and red lines indicate the REMA-DEM surface elevations before (29 September 2016) and after (1 October 2017) the 2017 GLOF event, respectively. The gray line with dots indicates the subglacial elevation from BedMachine^43^. The broken line indicates the floating condition of ice-dam, which satisfies the flotation criteria based on the ice-sheet and lake-surface elevations on 29 September 2016.

This flotation hypothesis could be investigated by analyzing the ice-surface, ice-sheet bed, and lake-surface elevations. The elevation difference between the lake level and flotation level, $\Delta z$, is denoted as follows:

1 $$\Delta z = h_{f} - h_{l} = h_{b} + \frac{{\rho_{i} }}{{\rho_{w} }}\left( {h_{s} - h_{b} } \right) - h_{l}$$

where $\rho_{i}$ and $\rho_{w}$ are the ice and water densities, respectively; and $h_{l}$, $h_{s}$, $h_{b}$, and $h_{f}$ are the lake-surface, ice-surface, ice-sheet bed, and flotation threshold elevations, respectively. We employed $h_{b}$ from the BedMachine dataset^43^ because it is currently the only available bed-elevation dataset that covers our study region. The regions where $\Delta z$ ≤ 0 indicate that the potential for flotation exists when the subglacial environment is hydrologically connected to the lake. The spatial distribution of $\Delta z$ highlights that the lake level is below the flotation threshold around LKI, thereby ensuring that the lake is dammed by the ice sheet (Fig. 4a). The area to the south of the LKI–EAIS boundary is the closest to the flotation criterion, whereas the other areas along the ice-dammed boundary rapidly increase to large positive $\Delta z$ values, such that these areas would prohibit flotation (Fig. 4b).

**Figure 5:**
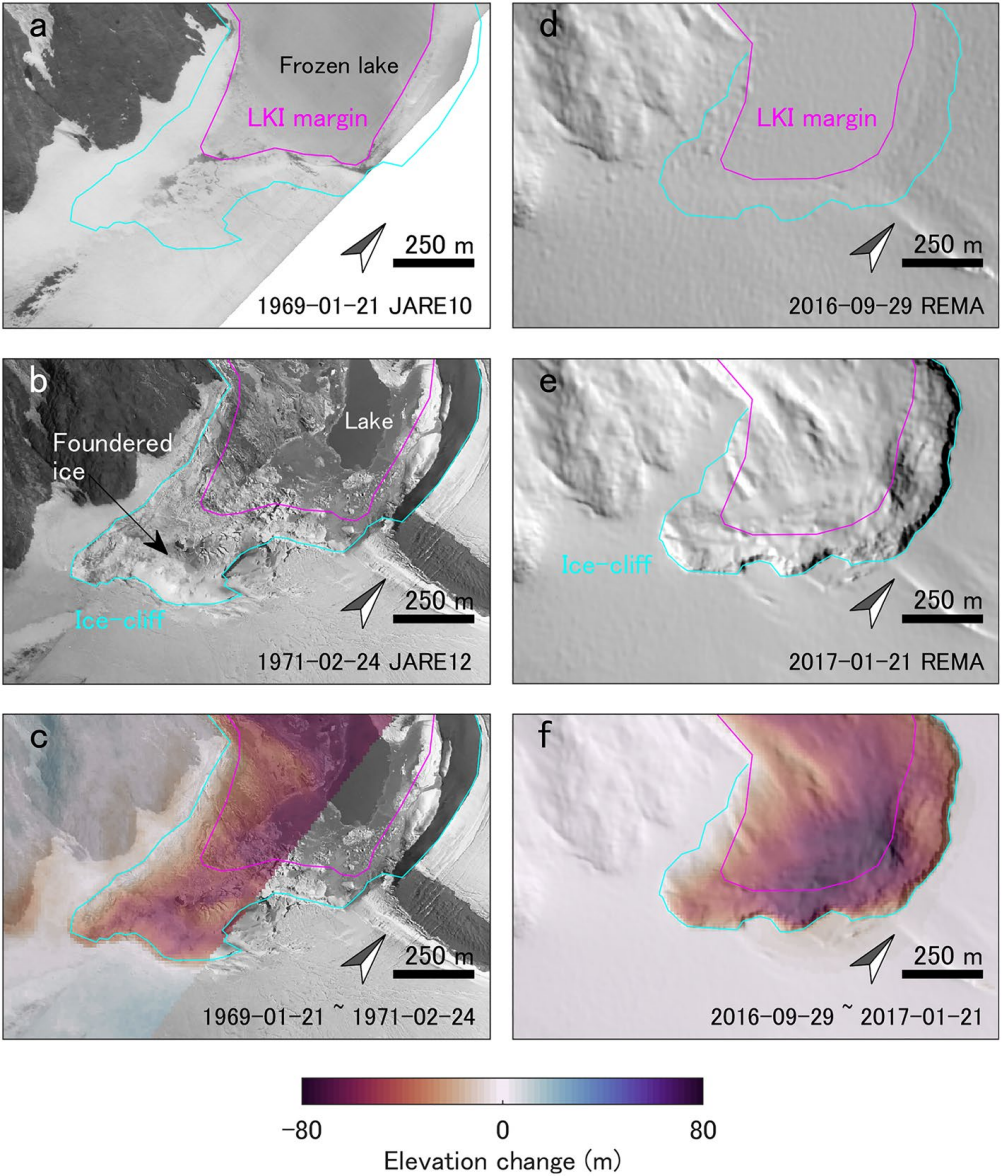
Comparison of the surface features at LKI and the surrounding EAIS. Changes in the surface features and elevation during the (**a**–**c**) 1969–1971 GLOF and (d–f) 2017 GLOF events. Orthoimages on (**a**) 21 January 1969 and (**b**) 24 February 1971. (**c**) Surface-elevation change between 21 January 1969 and 24 February 1971. Shaded relief from REMA on (**d**) 29 September 2016 and (**e**) 1 October 2017. (**f**) Surface elevation change between the 29 September 2016 and 1 October 2017 REMAs. Magenta lines denote the LKI margins prior to the GLOF events, and cyan lines denote the edge of the ice sheet after the GLOF events.

The southerly route (approximately parallel to profile BC in Fig. 4b) is the most likely drainage route. However, there is a large discrepancy between the bed elevation from BedMachine^43^ and floating condition of ice-dam that calculated from the surface elevations of the ice sheet and lake, which implies that the available bed-elevation data do not satisfy the flotation threshold. A > 10-m increase in the lake level is required to reach flotation based on the available bed-elevation constraints. Nevertheless, the actual threshold is less than ~ 20 m due to the effect of the gradual opening of the subglacial seal^44^. This assumption is based on the idea of ice deformation on the lakeward side as a “cantilever” effect, whereby the accumulated lake water induces buoyancy-driven uplift of the glacier-ice. Furthermore, the errors in the bed-elevation dataset introduce uncertainties in the exact location of the drainage route owing to the coarse resolution (500 m) and large uncertainties (100–1000 m) of the employed bed-elevation dataset. We note that these large uncertainties are due to a lack of measurements across the study region and the fact that the BedMachine dataset^43^ is designed for large-scale studies that incorporate either the entirety of Antarctica or regional catchments. We observed > 100 m of ice-cliff retreat (Fig. 5a and b) and subsidence of the ice-sheet surface and collapsed ice near the lake (Fig. 5c) after the 1969–1971 event. These features are similar to the ice-sheet surface features that have been observed after subglacial drainage at the Antarctic Peninsula^27^. The observed retreat following the 2017 event was smaller than that following the 1969–1971 event, and there was no clear subsidence/collapse feature in the ice-sheet surface (Fig. 5d–f). Nevertheless, the surface subsidence that was observed following the 1969–1971 event occurred in a region that did not satisfy the flotation criterion (Fig. 4), which implies uncertainties in the bed-elevation dataset^43^ for this region. Furthermore, other ice-dammed lakes located on the northeastern side of LKI showed neither changes in area nor water overflow. This implies that the northern drainage route was not activated, which supports a southerly drainage route. Therefore, we conclude that the LKI GLOFs occurred owing to flotation of the ice dam, with the southerly route serving as the most likely drainage pathway.

The 2017 PALSAR-2 images revealed that the 2017 LKI GLOF event occurred during the austral winter (Fig. 1e and f). Although flotation of the ice dam is the most likely mechanism of the LKI GLOFs, it is unclear what triggered the breach of the water-pressure threshold, which then allowed the formation of a drainage pathway beneath the ice dam. Ice/snow meltwater could contribute to the increase in lake volume during the melt season in other regions^42, 45^. However, the surface temperature was always below 0 °C across the study region, and no significant snowfall events were observed at Syowa Station during the April–May 2017 period (Supplementary Fig. 3). Additional water inputs from the subglacial environment, such as the drainage from either a nearby subglacial lake or a developed subglacial hydrological system, may assist in reaching the water-pressure threshold for LKI drainage. However, there are no known subglacial lakes near LKI, and the subglacial thermal regime near LKI is unknown.

The volume of LKI increased by 1.7 × 10^6^ m^3^ a^−1^ during the 1971–2017 period and 2.5 × 10^6^ m^3^ a^−1^ during the 2017–2021 period, despite such a limited supply of liquid water via atmospheric processes. Furthermore, there were no distinct signals from summer meltwater input in the surface-elevation and lake-volume time series (Fig. 3). These results imply a constant water input from the subglacial hydrological network on a year-round, such that the subglacial water pressure at the ice dam can reach the threshold via constant subglacial discharge during the austral winter, even if there are no other water inputs into LKI. Although the water supply is independent of the atmospheric conditions, it does depend on the geothermal flux and subglacial catchment geometry, which are basically invariable. The presence of an active subglacial network has been suggested by direct observations of submarine groundwater discharge in Lützow-Holm Bay^46^ and an equipotential hydrostatic model^47^. Nevertheless, the actual trigger of the LKI GLOFs is not constrained at this stage because the subglacial environment is generally frozen around the periphery of EAIS^48^.

Our long-term observations provide the first evidence of repeated GLOFs from an ice-marginal lake in Antarctica (Figs. 2 and 3). Although the lake-volume time series implies a similar pattern to those of cyclic GLOFs, the cyclicity of the LKI GLOFs is inconclusive due to the small number of the events. If we apply the simple assumption that the time between the two GLOFs defines the periodicity of LKI GLOFs, then LKI possesses a GLOF periodicity of ~ 50 years, which is longer than the GLOF periodicities at ice-marginal lakes in other regions^36, 49, 50^. Meltwater availability and/or rainfall are key factors that contribute to these differences in GLOF periodicity, with local subglacial hydrological processes and the physical properties (temperature, softness, and permeability) of the ice damming the lake, which are currently unconstrained for LKI and the surrounding area. Recent increases in rainfall and intensive melting along the periphery of EAIS, which have been attributed to changes in atmospheric conditions^51, 52^, may both accelerate the occurrence of the next LKI GLOF event and increase the frequency of future GLOF events at LKI and other ice-marginal lakes. Nevertheless, there are limited examples of GLOFs associated with ice-dammed lakes in Antarctica, and the time series of these events are too short to distinguish their periodicity^23–26^. Thus, continued monitoring of the ice-dammed lakes in Antarctica is needed to further discuss the potential periodicity of GLOFs and how a changing global climate may impact this periodicity.

### Implications for AIS hydrology and the surrounding environment

The large-scale GLOFs that have been observed from ice-marginal lakes in Greenland^53^ also highlight the need to monitor ice-marginal lakes in Antarctica, as continued warming will alter AIS dynamics and atmospheric conditions in the coming decades and centuries^54, 55^. Detailed mapping of the ice-dammed and ice-marginal lakes around AIS is recommended to assess the contribution of such lakes as potential GLOF sources, as has been done for the Greenland Ice Sheet^56^. Furthermore, our results imply a possible subglacial water input into the lake, even during the austral winter. Subglacial water has been observed beneath the Greenland Ice Sheet and is recognized as a key factor in modulating ice movement^57, 58^. In fact, substantial accelerations in glacier flow have been observed after lake drainage in Greenland^59, 60^ and Alaska^61^. Although the subglacial hydrology of these glacierized regions remains unknown, these examples imply that Antarctic GLOFs could potentially trigger accelerations in ice flow. Therefore, more detailed measurements of the subglacial topography around the periphery of AIS are required to both elucidate potential GLOF triggers and map where ice-marginal lakes may form owing to the future retreat and/or thinning of AIS, as has been conducted in other glacierized regions^62^.

Our results also imply the sporadic influx of freshwater to the ocean. However, our understanding of the freshwater supply from AIS to the surrounding ocean is currently limited. Recent modeling studies have shown that there is a limited contribution to the total basal melt of the ice shelves, with enhanced melt in sub-ice-shelf channels^63, 64^ due to subglacial freshwater discharge. Although the amount of freshwater that is discharged via LKI GLOF events is much less than the discharge in these modeling studies^63, 64^, a sporadic freshwater pulse could promote a similar enhanced melt effect if the water discharges toward ice shelves. Nutrients and sediment transport are also expected owing to the freshwater transport, which may have an influence on nearby ocean ecosystems considering changes in the chemical properties of Antarctic ice-marginal lakes after GLOF events^13^. Although constraints on the subglacial discharge from AIS to the ocean are currently quite limited, such that the impact of this freshwater flux remains unknown, it is important to monitor such lake and freshwater transport to advance our understanding of this phenomenon along the periphery of AIS.

## Conclusion

In this study, we revealed the occurrence of repeated GLOFs from LKI, an ice-dammed lake in East Antarctica, during the 1962–2021 period. Several types of data (SfM-/satellite-DEMs, satellite altimetry, and a combination of the shoreline position and lake bathymetry) were utilized to quantify the changes in lake-surface elevation and lake volume. We estimated (8.6 ± 1.5) × 10^7^ and (7.1 ± 0.4) × 10^7^ m^3^ of water discharge during the 1969–1971 and 2017 GLOFs, respectively, which are two of the largest known GLOFs in Antarctica. Surface- and bed-elevation constraints have indicated that the GLOFs likely occurred via flotation of the ice dam, with water discharge occurring through a southerly drainage pathway from LKI. Furthermore, the 2017 GLOF occurred during the austral winter (April–May), thereby suggesting a year-round active subglacial hydrological network because there were no water inputs into LKI from surface processes.

Our results, coupled with the lack of subglacial constraints in the region, emphasize the importance of elucidating the subglacial drainage system of AIS and highlight that continuous monitoring of ice-marginal lakes may provide important insights into AIS subglacial hydrology. Radar and/or seismic surveys are required to provide more accurate and detailed constraints on the subglacial topography and internal structure of ice dams. Therefore, our results will encourage future monitoring of Antarctic ice-marginal lakes and additional observations of AIS hydrological processes.

## Methods

### Aerial photography and satellite imagery

We analyzed aerial photographs and optical satellite images to delineate the surface features across LKI. JARE has acquired aerial photographs across Lützow-Holm Bay since 1957, with aerial photographs taken across the Skarvsnes region during the austral summers of 1957, 1962, 1969, 1971, 1983, 1992, 1993, and 2000. We obtained these freely available photographs, which were scanned at 400 dpi, from the Geospatial Information Authority of Japan (GSI; https://www.gsi.go.jp/antarctic/06.html) for our time-series analysis. The spatial resolutions of the aerial photographs are 0.83–2.16 m. We also used optical satellite images acquired by the Landsat 1 Multispectral Scanner (MSS), Landsat 4 MSS/Thematic Mapper (TM), Landsat 5 MSS/TM, Landsat 8 Operational Land Imager (OLI), Sentinel-2 Multi Spectral Instrument (MSI), and Terra Advanced Spaceborne Thermal Emission and Reflection Radiometer (ASTER) (DatalistOpticalSatellite.xlsx). The spatial resolutions of these satellite images are 60 m for MSS, 30 m for TM, 15 m for OLI and ASTER, and 10 m for MSI. False-color composite images were constructed using the red, green, and near-infrared band images that were included in each product. Note that we used pansharpened false-color Landsat 8 images, which were constructed using the Landsat 8 panchromatic images in the QGIS software.

We analyzed both SAR and optical satellite imagery to investigate the intra-annual variations in the 2017 lake-surface features. We used two SAR images that were acquired on 13 April and 15 May 2017 by the Phased Array type L-band Synthetic Aperture Radar 2 (PALSAR-2) mounted on the Advanced Land Observing Satellite 2 (ALOS-2) satellite for this purpose. Radiometric calibrations, multi-looking (two looks and one look in the azimuth and range directions, respectively), speckle reduction via the Refined Lee filter, and ellipsoidal corrections were applied to the Level 1.1 data (single-look complex) using the freely available Sentinel Applications Platform (SNAP) software developed by the European Space Agency. A terrain correction was then applied using the REMA-composite DEM in SNAP.

The Landsat MSS and PALSAR-2 images possessed enormous horizontal geo-location errors (~ 2.4 km for MSS and ~ 300 m for PALSAR-2). We corrected these errors using the Georeferencer Plugin in the QGIS software and manually located ground control points (GCPs). Four GCPs were located within each image to determine their near-edge positions.

### DEM datasets

We used five types of DEMs for the Skarvsnes region to retrieve the surface-elevation changes across LKI: SfM-DEMs, a GSI-DEM, an ALOS-DEM, REMA-DEMs, and a TDX-DEM.

We applied the SfM tool in Metashape Pro version 1.7.1 software (©Agisoft, 2021) to the JARE aerial photographs to construct the SfM-DEMs. We first applied manual masking to eliminate the camera frames that were included in the periphery of the aerial photographs. We then followed the approach outlined in a previous study^33^ as follows. We first performed initial image matching, alignment, and three-dimensional (3-D) model reconstruction without any GCPs. We then extracted 53 GCPs from the 1993 DEM and orthoimage of the Skarvsnes region^33^ and manually located these GCPs on the aerial photographs (Supplementary Fig. 4). We subsequently performed the above-mentioned 3-D model reconstruction using the geo-located aerial photographs to construct a geo-located 3-D model. Finally, we constructed geo-located DEMs and orthoimages from the 3-D models. The spatial resolutions of the DEMs and orthoimages were 2.9–4.2 and 1.4–2.1 m, respectively. Note that the 1983 aerial photographs were not used for the DEM construction because the photographs over the Skarvsnes region were taken along a single flight path. We also did not use the 1957 images because they were oblique images, although they have enabled us to assess the state of LKI in 1957 (Supplementary Fig. 1).

The GSI-DEM was constructed from a pair of 2.5-m resolution stereo images that were acquired on 18 January 2009 by the ALOS/Panchromatic Remote-sensing Instrument for Stereo Mapping (PRISM). GSI performed the DEM construction, which possessed a 10-m spatial resolution. The vertical accuracy of the GSI-DEM was evaluated to be 3.82 m based on a comparison of static geodetic survey constraints and the GSI-DEM elevations at a series of outcrops across the Skarvsnes region^33^. We also applied the photogrammetric tools in the Ames Stereo Pipeline software^65^ version 2.7.0 to the nadir and backward stereo-pair ALOS/PRISM images that were acquired on 19 November 2007 to construct a DEM.

We used the REMA-DEMs^34, 35^ and other DEMs to investigate the recent surface-elevation changes across LKI. The REMA-DEMs were constructed by applying the photogrammetric tools in the SETSM software^66^ to panchromatic stereo images acquired by the WorldView-1/-2/-3 and GeoEye-1 satellites. We used 22 strip REMA-DEMs (version 4.1) to retrieve the surface-elevation changes across LKI. Each strip REMA-DEM was constructed from a pair of satellite stereo images and, therefore, possessed a precise acquisition date. The spatial resolutions of the original distributed strip REMA-DEMs are 2 m. We used the composite REMA-DEM as the reference DEM in this study because the composite REMA-DEM was constructed by mosaicking the strip REMA-DEMs and possessed a reliable vertical coordinate^34^. The spatial resolution of the composite REMA-DEM was 10 m, and the vertical accuracy was reported to be < 1 m^34^.

A TDX-DEM was generated from X-band SAR data that were acquired simultaneously by the TerraSAR-X add-on for Digital Elevation Measurement (TanDEM-X) and TerraSAR-X on 8 June 2017 during the bistatic operation mode. We applied interferometric SAR processing to the SAR data pair and then converted the obtained interferometric phase of each pixel to a surface height with a precisely determined perpendicular baseline length (Bp) between the two satellites. We used GCPs from AW3D30 version 3 (https://www.eorc.jaxa.jp/ALOS/en/dataset/aw3d30/aw3d30_e.htm) to determine the precise orbits during the Bp estimation. The generated TDX-DEM was processed using the GAMMA software version 20220630.

### DEM corrections

We applied the following corrections to the DEMs to remove the complex errors that are often included in DEMs^67^. We followed the method proposed by Nuth and Kääb^67^, whereby we removed the horizontal shifts, elevation-dependent biases, and sensor-dependent biases (along-/across-satellite track biases) from the original DEMs. We only applied horizontal shift and elevation-dependent bias corrections to the SfM-DEMs because the along-/across-satellite tracks were not determined for the SfM-DEMs. We then subtracted the second-order surfaces from the SfM-DEMs to remove any doming/bowl errors that are typically included in SfM-DEMs^68, 69^. The surfaces were fitted via the least-squares method to the difference between each SfM-DEM and the reference DEM. Note that the TDX-DEM includes long-wavelength noise because the TDX-DEM was constructed via SAR interferometry of the TanDEM-X and TerraSAR-X satellites. We subtracted the noise by applying the least-square method to estimate the second-order surfaces after the elevation-dependent bias-removal correction. We corrected the ALOS-DEM by removing the horizontal shifts and elevation-dependent biases, and the GSI-DEM, SfM_93_-DEM, and strip REMA-DEMs by removing the vertical offsets. Finally, all of the DEMs were aligned to the grid of the reference DEM to yield DEMs with a consistent 10-m spatial resolution (Supplementary Figs. 4 and 5).

The above corrections were performed on DEMs that were set to the reference DEM elevation over the outcropping bedrock across the Skarvsnes region (yellow region in Supplementary Fig. 6b), where the elevation differences among the various constructed DEMs and reference DEM were assumed to be negligible. We first subtracted the differences between each DEM and the reference DEM over the outcropping bedrock and then applied elevation-dependent, sensor-dependent, and doming/bowl corrections that were estimated via least-squares fitting to the bedrock-corrected DEMs. We excluded the regions across LKI where the slope was steep (> 15°) relative to the reference DEM during the corrections. We also removed any pixels in a given DEM that possessed differences of more than three standard deviations between the corrected DEM and the reference DEM. We then took the standard deviations of the difference between each corrected DEM and the reference DEM to be the uncertainty of that DEM. The elevation uncertainties in the corrected SfM-DEMs, ALOS-DEM, TDX-DEM, and REMA-strip-DEMs were 2.7–5.9, 2.7, 5.0, and 1.0–6.0 m, respectively (Supplementary Table 1).

### Lake-surface elevation and lake volume

The lake-surface elevation was derived from the DEMs as the approximately constant elevation value across LKI, such as the 162 m above sea level (a.s.l.) contour in Fig. 1c. We assumed that the elevation uncertainty was equal to the vertical uncertainty in the DEMs. We also used ice-surface elevation measurements from the ICESat-2/ATLAS ATL06 product^70^ version 5. These consisted of two ICESat-2 tracks that crossed the lake surface on 28 February 2019. The elevation values inside the lake area were averaged to obtain the lake-surface elevation (Supplementary Fig. 7), and the standard deviation was taken to be the measurement uncertainty.

We also estimated the lake-surface elevation using the optical satellite imagery and the 1 October 2017 REMA-DEM (REMA_empty_). REMA_empty_ was acquired shortly after the lake drainage, when the lake level was low; therefore, it should represent the lake-bed geometry (Supplementary Fig. 8). We assumed that the REMA_empty_ contour lines in the lake area represented the lake extent at an arbitrary elevation, such that the lake-surface elevation was determined by comparing the REMA_empty_ contour lines and surface elevations in the optical satellite images. The elevation of the best-fit contour line was defined as the lake-surface elevation for the date when the satellite image was acquired. We iterated this procedure to determine the upper and lower elevation bounds, which were often different based on the features along the lake margin (Supplementary Fig. 9). The range between the upper and lower bounds was assumed to be the measurement uncertainty. We used the false-color composite images that were constructed from the Landsat 5–8, Sentinel-2, and ASTER imagery (DatalistOpticalSatellite.xlsx) for this procedure.

We calculated the relative lake water volume $dV$ from the elevation difference between each DEM and the reference DEM as follows:

2 $$\begin{array}{*{20}c} {dV\left( t \right) = \mathop \int \nolimits_{lake} dh\left( t \right) = \overline{dh} \left( t \right) \cdot A_{lake} \left( t \right)} \\ \end{array}$$

where $dh\left( t \right)$ is the relative height of the lake surface at time $t$ relative to the reference DEM, and $A_{lake} \left( t \right)$ is the transient lake area, which is estimated from REMA_empty_. $\overline{dh} \left( t \right)$ is the mean elevation difference relative to the reference DEM over the lake. We also used the lake-surface elevations that were retrieved from the ICESat-2 data to calculate the relative lake volume from pseudo DEMs, whereby the two-dimensional lake-surface elevations from inside the known bounds of LKI were used to infer $dh\left( t \right)$ and $A_{lake} \left( t \right)$ from REMA_empty_. The uncertainty in the lake-volume change $\sigma_{dV}$ is assumed to be:

3 $$\sigma_{dV} \left( t \right) = \sigma_{dh\left( t \right)} \cdot A_{lake} \left( t \right)$$

where ${\sigma }_{dh\left(t\right)}$ is the uncertainty in the elevation difference. Note that we subtracted the lake-volume change owing to the migration of the ice-sheet margin because the LKI–EAIS boundary was recognized.

### Flotation potential

We investigated the possibility of the LKI ice dam reaching flotation. We used Eq. (1) to test this hypothesis under the assumption that the ice where $\Delta z$ < 0 can go afloat when the subglacial environment is hydrologically connected to the lake due to the power balance. The local bed elevation was taken from BedMachine^43^ version 3.

### Supplementary Information

Supplementary Information 1.

Supplementary Information 2.

Supplementary Information 3.

## Data Availability

The REMA-DEMs were downloaded using the REMA Strip DEM extent index and REMA Mosaic DEM extent index (https://www.pgc.umn.edu/data/rema/); each URL is summarized in Supplementary Data S2 (DatalistREMA.xlsx). The Landsat images were downloaded from Earth Explorer (https://earthexplorer.usgs.gov/). The Sentinel-2 images were downloaded from https://scihub.copernicus.eu/. The ASTER images were downloaded from https://gbank.gsj.jp/madas/. The SfM-DEMs constructed in this study, lake-surface elevations, and relative volume time series for LKI are available from 10.17592/001.202311202 and 10.17592/001.2023112001, respectively. The TanDEM-X and TerraSAR-X data were downloaded from https://eoweb.dlr.de/egp/. The precipitation data for Syowa Station were downloaded from https://www.data.jma.go.jp/gmd/risk/obsdl/.

## Code availability

The map of the study site (Fig. 1a) was generated using open-source GIS software (QGIS 3.22). The other figures were generated using MATLAB 2023a (https://mathworks.com/products/matlab.html). The MATLAB scripts were written using standard MATLAB functions and functions that are included in M_Map^71^ version 1.4 m, which is a freely available toolbox.
