# Supplementary material for: Outbursts from an ice-marginal lake in Antarctica in 1969–1971 and 2017, revealed by aerial photographs and satellite data
Source: Sci Rep. 2023 Nov 27;13:20619. doi: 10.1038/s41598-023-47522-w (PMC10682390; doi:10.1038/s41598-023-47522-w)
Supplement: Supplementary file 1 — Supplementary Information 1. [file 41598_2023_47522_MOESM1_ESM.pdf]

Outbursts from an ice-marginal lake in Antarctica in 1969–1971 and 2017, revealed by aerial photographs and satellite data

Shuntaro Hata[1,2*], Moto Kawamata[3], and Koichiro Doi[4,5]

1. Institute of Low Temperature Science, Hokkaido University, Sapporo, Japan
2. Creative Research Institution, Hokkaido University, Sapporo, Japan
3. Civil Engineering Research Institute for Cold Region, Public Works Research Institute, Sapporo, Japan
4. National Institute of Polar Research, Tokyo, Japan
5. The Graduate University for Advanced Studies (SOKENDAI), Japan

*To whom correspondence: Shuntaro Hata (hata@lowtem.hokudai.ac.jp)

Contents of this file

Supplementary Figure 1–9

Supplementary Table 1

Additional Supporting Information (Files uploaded separately)

Datasets S1 to S2

Introduction

The auxiliary materials for "Outbursts from an ice-marginal lake in Antarctica in 1969–1971 and 2017, revealed by aerial photographs and satellite data".

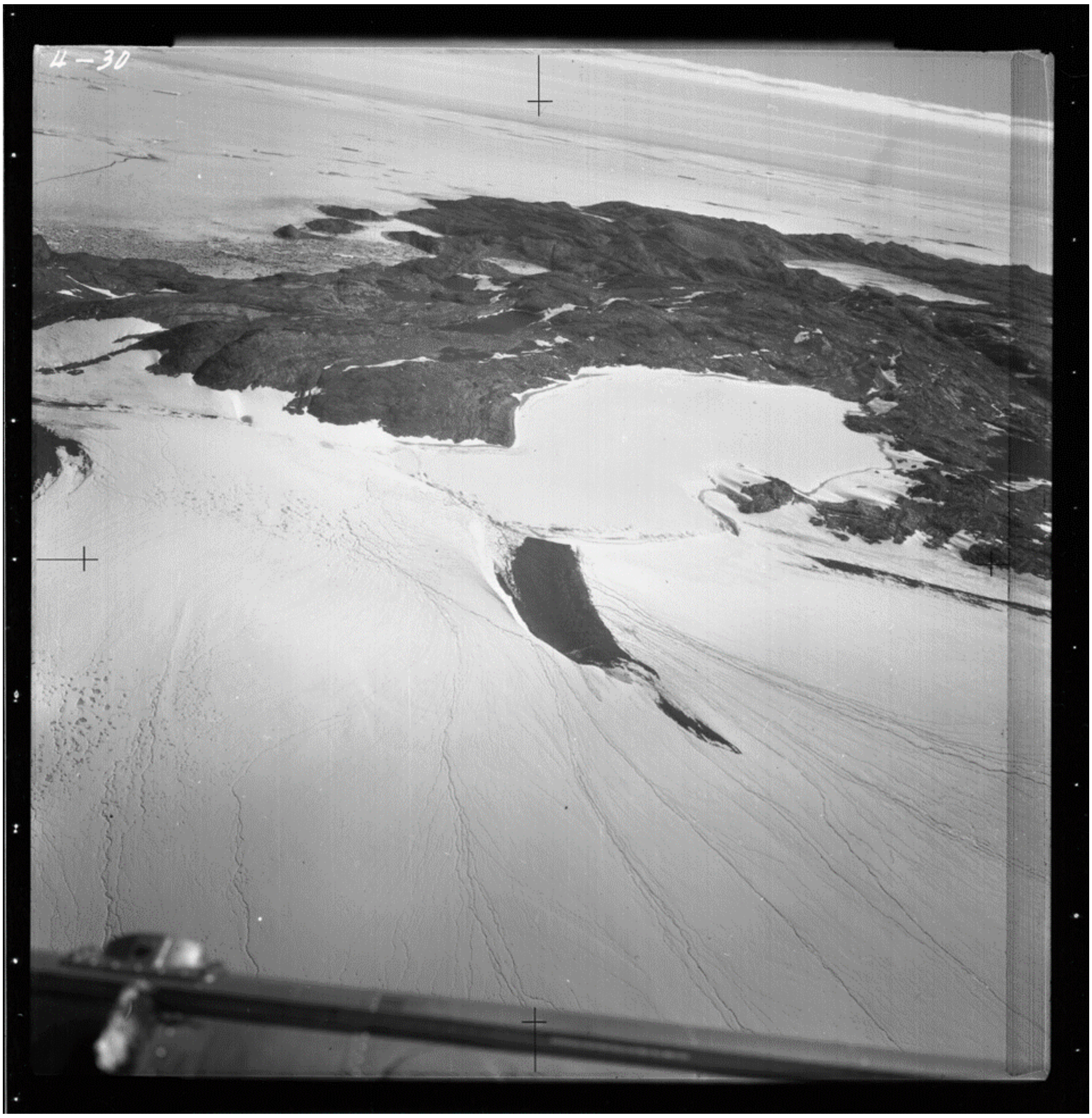

Supplementary Figure 1. Aerial photograph in 1957-01-18.

The first photograph taken in JARE1 campaign which captures LKI. The image enables us to see near-fully stored situation.

(Image source: https://www.gsi.go.jp/antarctic/06/01th_45th/LutzowHolmBayEastCoast_1AV-II_01.html#1AV-II-1-122-30).

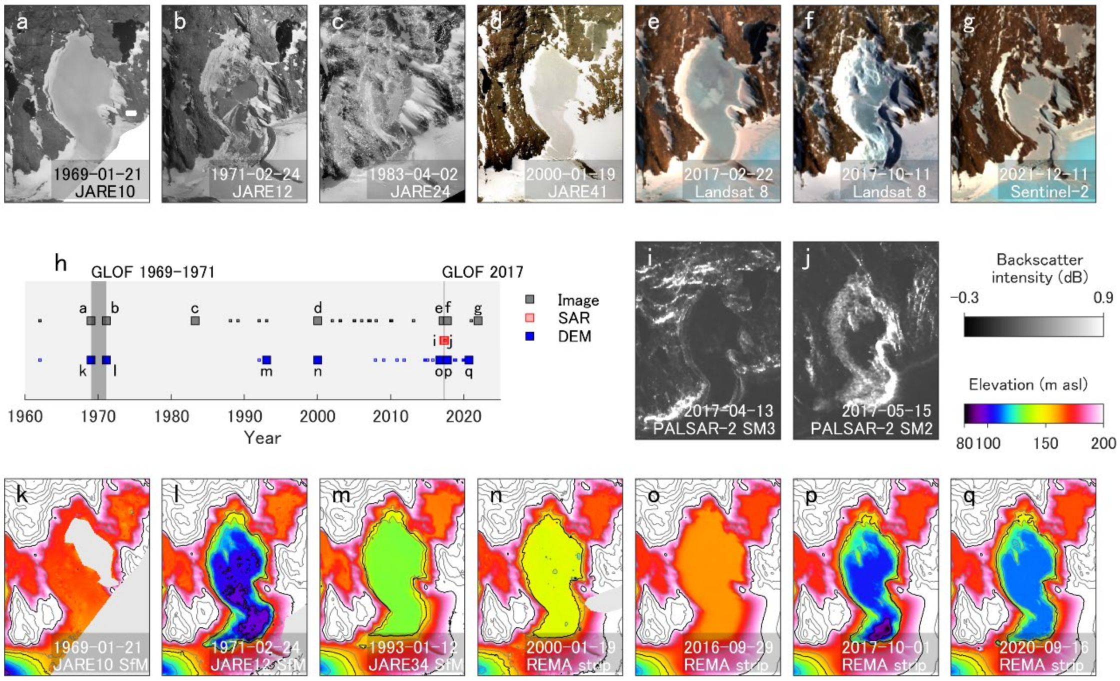

Supplementary Figure 2. Summary of variation in surface features and elevation of LKI.

(a–d) orthoimages, (e–g) optical satellite images, (i, j) backscatter intensity image, and (k–q) surface elevation of LKI. The dates are represented in the lower part of each image. (h) time slots of the other panels. Small boxes indicate other images/DEMs not represented in this figure but used for the analysis.

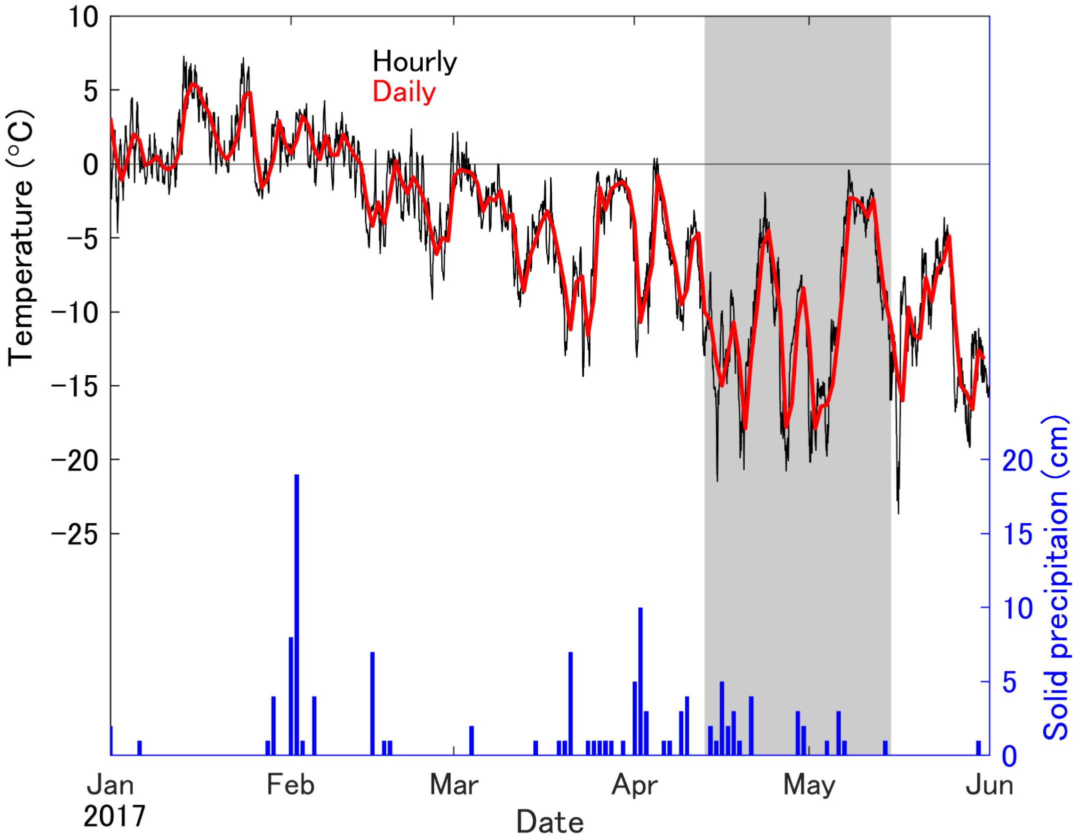

Supplementary Figure 3. Weather conditions around the second event.

Timeseries of hourly (black) and daily (red) temperature measured at Kizahashi-hama AWS (Supplementary Figure 6b) (Kudoh et al., 2021). Daily solid precipitation observed at Syowa Station (blue) is also shown. Grey hatch indicates the period of the second event determined by PALSAR-2 backscatter intensity images.

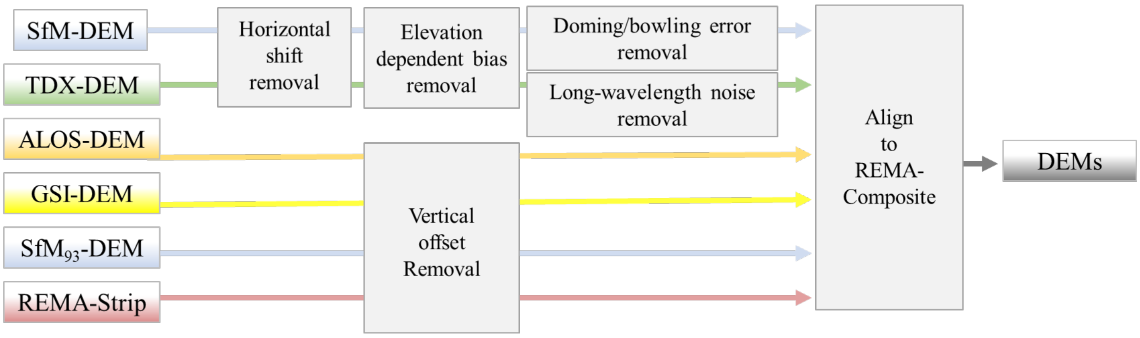

Supplementary Figure 4. Workflow of the DEM correction.

Each DEM was applied selected methods. Thereafter, all DEMs were aligned to the grid of REMA-composite DEM (reference DEM).

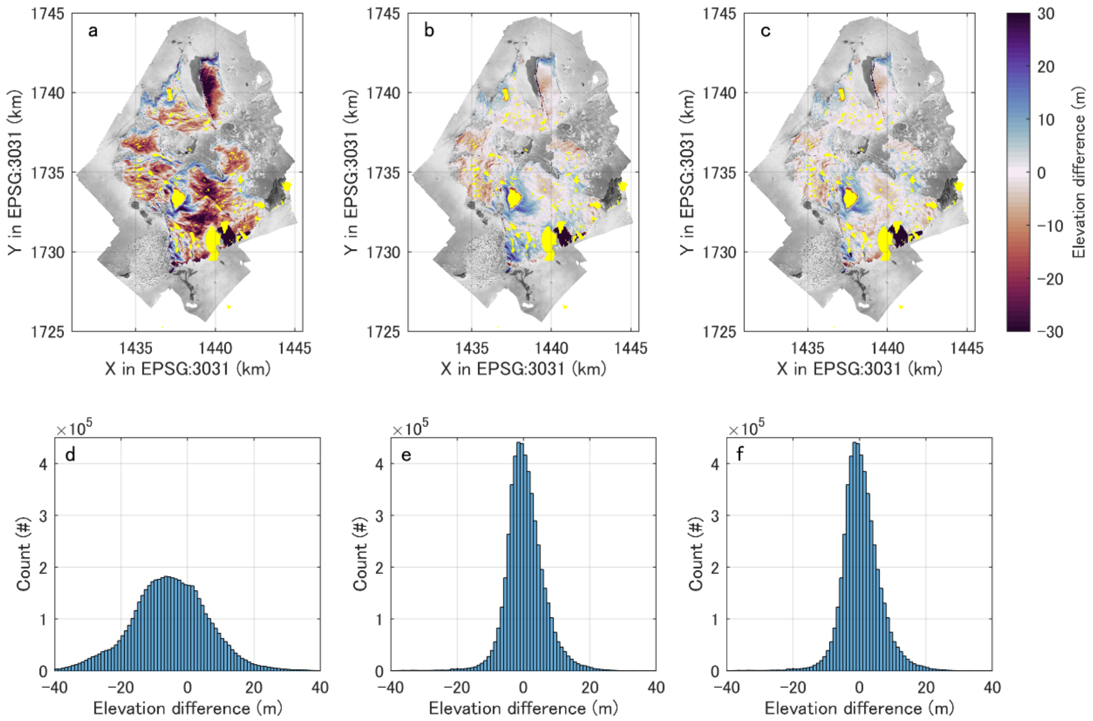

Supplementary Figure 5. Example of DEM correction for SfM-DEM.

a) Elevation difference of SfM-DEM from 1962 images and reference REMA-composite DEM. Yellow regions indicate lakes in Skarvsnes. The background image is the orthomosaic of 1962 constructed by SfM procedures. b, c) Same figure as a) but difference between reference DEM and DEM after applying (b) the correction method of Nuth and Kääb (2011) and (c) the doming/bowl removal. d–f) Histograms of the elevation difference between reference DEM and (d) original SfM-DEM, SfM-DEM applied the correction method of Nuth and Kääb (2011), and SfM-DEM applied the doming/bowl removal, respectively.

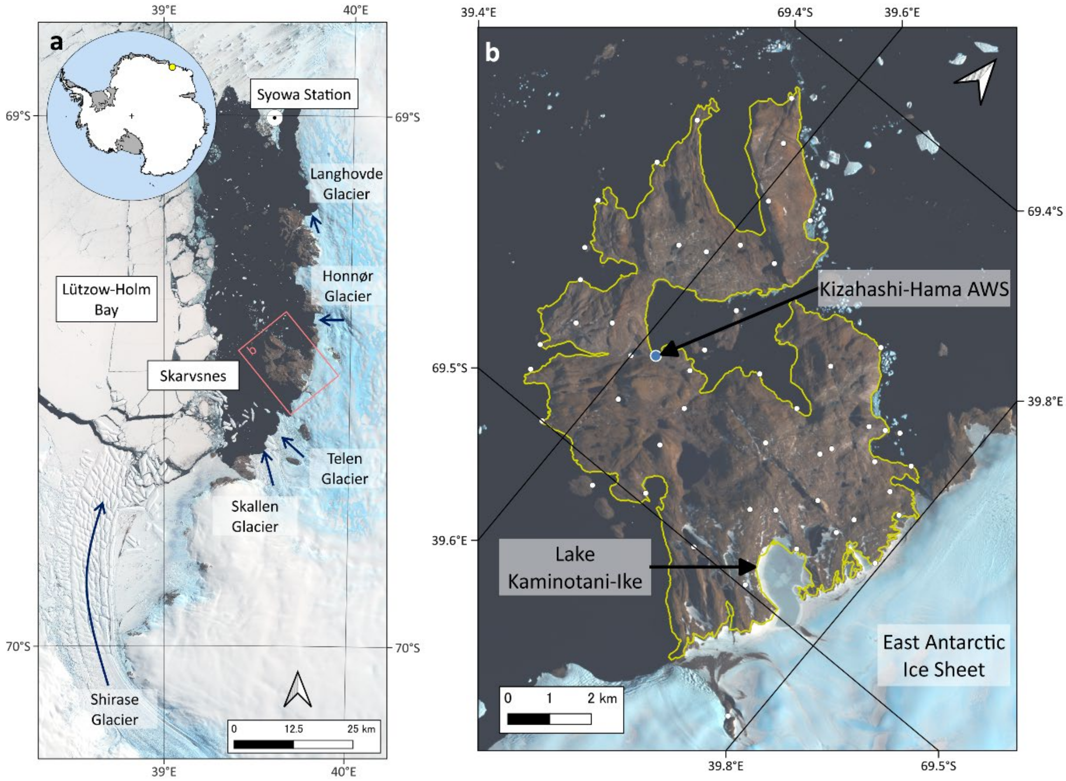

Supplementary Figure 6. Map of the Skarvsnes.

a) Satellite image showing an overview of the Lützow-Holm Bay, taken on 17 February 2013. Outlet glaciers of EAIS in this region are also shown with the direction of their flow. Box indicates the location of (b). b) Close-up view of Skarvsnes outcrop. White dots and yellow region indicate GCPs used for constructing SfM-DEMs and region used for correction and evaluation for DEMs.

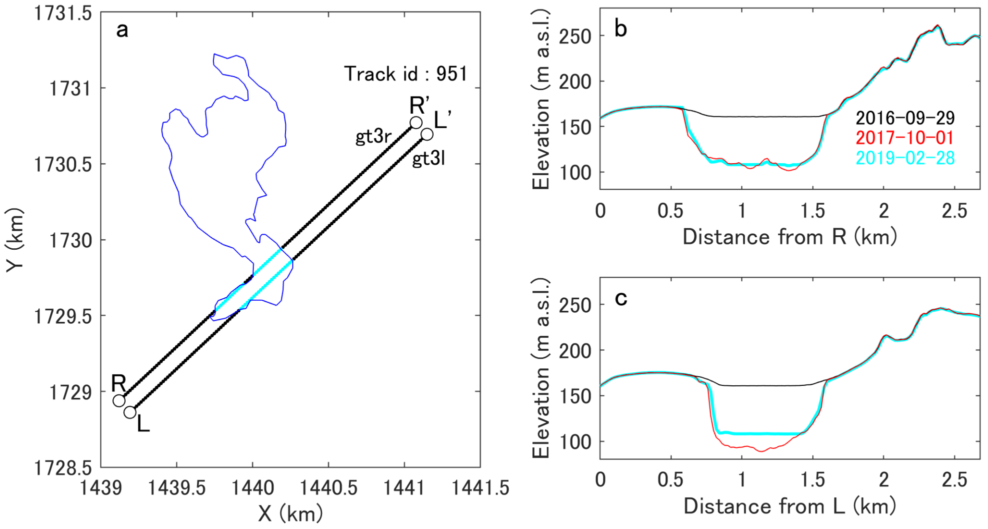

Supplementary Figure 7. Surface elevation from ICESat-2 data.

a) The locations of LKI and ICESat-2 tracks on 2019-02-28. Blue line indicates outline of LKI on 4 January 2021. b, c) surface elevation along profiles of RR' and LL'. Location of the profiles are shown in (a).

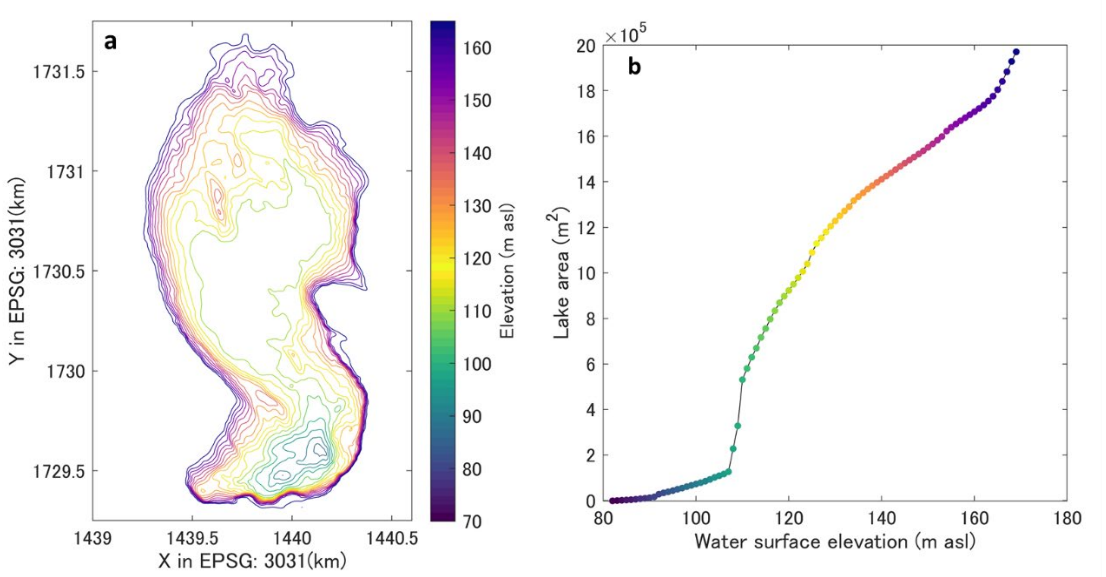

Supplementary Figure 8. DEM of LKI immediately after the drainage in 2017.
a) Contour lines of REMA on 2017-10-01 of LKI. b). The surface area variation with certain surface elevation of the lake.

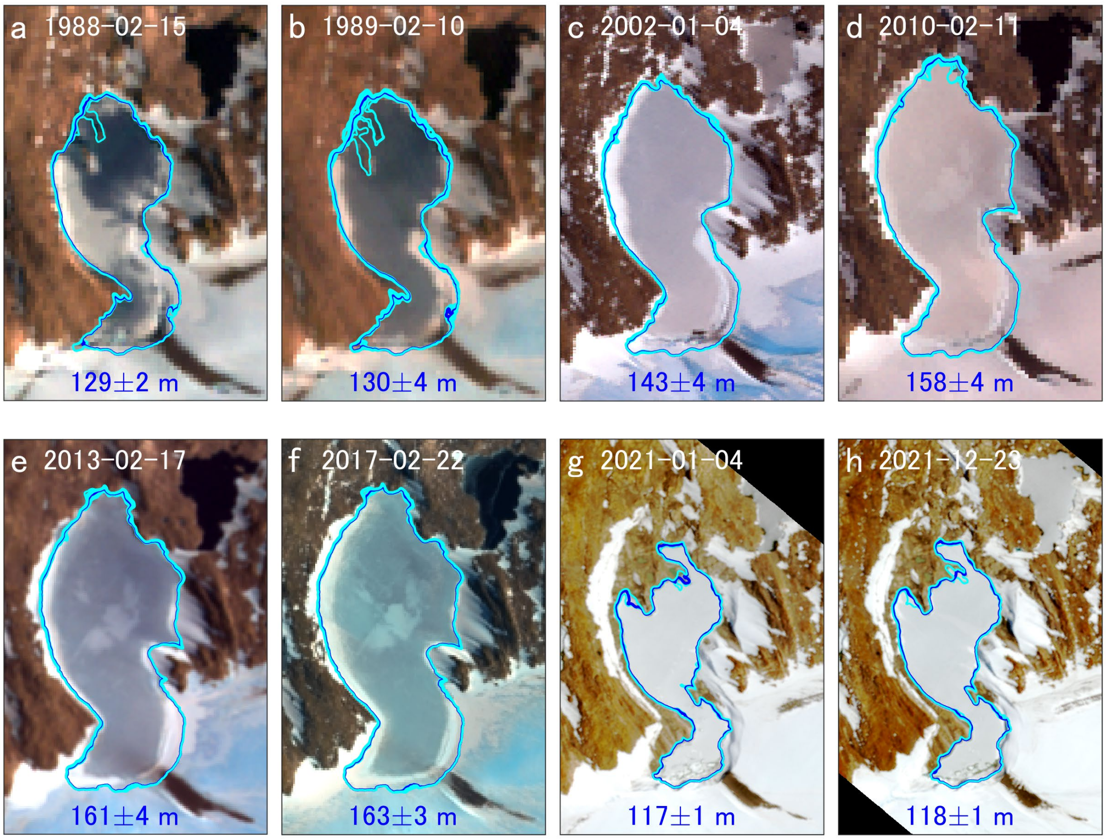

Supplementary Figure 9. Example of the lake surface elevation estimation from optical satellite images.

Blue lines indicate ones of the contour lines of the DEM on 1st October 2017 (Supplementary Figure 3a). The Best-fit contour line was determined to lake surface elevation to the background images, and cyan lines around represent the uncertainty of this method. The estimated surface elevation is represented in the lower part of each panel.

Supplementary Table 1. DEMs used for lake surface elevation measurement.

Name	Date	Uncertainty (m)	Reference
SfM-DEM	1962-01-22	±4.0	This study
	1969-01-21	±6.0	This study
	1971-02-24	±5.2	This study
	1992-01-20	±5.9	This study
	1993-01-12/17	±3.2	Kawamata et al. (2021)
	2000-01-19	±2.7	This study
ALOS-DEM	2007-11-19	±2.7	This study
GSI-DEM	2009-01-18	±3.8	Kawamata et al. (2021)
REMA	2010-11-23	±1.5	Howat et al. (2022)
	2011-11-11	±1.5	Howat et al. (2022)
	2014-09-10	±1.4	Howat et al. (2022)
	2014-09-12	±6.0	Howat et al. (2022)
	2014-09-16	±1.4	Howat et al. (2022)
	2014-09-21	±1.4	Howat et al. (2022)
	2014-09-23	±1.3	Howat et al. (2022)
	2015-01-30	±1.3	Howat et al. (2022)
	2015-10-12	±1.5	Howat et al. (2022)
	2016-09-29	±1.2	Howat et al. (2022)
	2017-01-21	±1.4	Howat et al. (2022)
TDX-DEM	2017-06-08	±5.0	This study
($REMA_{empty}$)	2017-10-01	±1.9	Howat et al. (2022)
	2017-11-14	±1.6	Howat et al. (2022)
	2018-02-22	±1.2	Howat et al. (2022)
	2018-09-15	±2.1	Howat et al. (2022)
	2018-11-13	±1.2	Howat et al. (2022)
	2018-11-22	±2.6	Howat et al. (2022)
	2019-12-04	±1.0	Howat et al. (2022)
	2019-12-06	±1.1	Howat et al. (2022)
	2020-01-24	±2.7	Howat et al. (2022)
	2020-03-03	±1.1	Howat et al. (2022)
	2020-09-16	±1.4	Howat et al. (2022)

Reference for Supplementary Information

Kudoh, S., Y. Tanabe, K. Hayashi, M. Kida, N. Fujitake, M. Uchida, and S. Imura. Meteorological data from ice-free areas in Yukidori Zawa, Langhovde and Kizahashi Hama, Skarvsnes, and Skallen on Sôya Coast, East Antarctica during January 2017 - December 2018. Arctic Data archive System (ADS), Japan, 2021. https://doi.org/10.20575/00000022

Nuth, C., and A. Kääb, Co-registration and bias corrections of satellite elevation data sets for quantifying glacier thickness change, The Cryosphere, **5**, 271–290, (2011), https://doi.org/10.5194/tc-5-271-2011.
